# Ceramicines M–P from *Chisocheton ceramicus*: isolation and structure–activity relationship study

**DOI:** 10.1007/s11418-017-1109-2

**Published:** 2017-08-18

**Authors:** Alfarius Eko Nugroho, Akiyo Hashimoto, Chin-Piow Wong, Hiromasa Yokoe, Masayoshi Tsubuki, Toshio Kaneda, A. Hamid A. Hadi, Hiroshi Morita

**Affiliations:** 1 Faculty of Pharmaceutical Sciences, Hoshi University, Ebara 2-4-41 Shinagawa-ku, Tokyo, 142-8501 Japan; 2 Department of Chemistry, Faculty of Science, University of Malaya, 50603 Kuala Lumpur, Malaysia

**Keywords:** Limonoids, Ceramicines, *Chisocheton ceramicus*, Lipid-droplet accumulation inhibition

## Abstract

Ceramicines are a series of limonoids which were isolated from the bark of Malaysian *Chisocheton ceramicus* (Meliaceae) and show various biological activities. Ceramicine B, in particular, has been reported to show a strong lipid droplet accumulation (LDA) inhibitory activity on a mouse pre-adipocyte cell line (MC3T3-G2/PA6). With the purpose of discovering compounds with stronger activity than ceramicine B, we further investigated the constituents of *C. ceramicus*. As a result, from the bark of *C. ceramicus* four new ceramicines (ceramicines M–P, **1**–**4**) were isolated, and their structures were determined on the basis of NMR and mass spectroscopic analyses in combination with NMR chemical shift calculations. LDA inhibitory activity of **1**–**4** was evaluated. Compounds **1**–**3** showed LDA inhibitory activity, and **3** showed better selectivity than ceramicine B while showing activity at the same order of magnitude as ceramicine B. Since **3**, which possess a carbonyl group at C-7, showed better selectivity than **5**, which possess a 7α-OH group, while showing activity at the same order of magnitude as **5**, we also investigated the effect of the substituent at C-7 by synthesizing several derivatives and evaluating their LDA inhibitory activity. Accordingly, we confirmed the importance of the presence of a 7α-OH group to the LDA inhibitory activity.

## Introduction

Ceramicines are a series of limonoids which were isolated in our previous phytochemical study on the bark of Malaysian *Chisocheton ceramicus* (Meliaceae). To date, 12 compounds were known, ceramicines A–L [1–4]. Ceramicines have been reported to show cytotoxic activity against a murine leukemia cell line (P388), potent anti-plasmodial activity against *Plasmodium falciparum* 3D7, lipid droplet accumulation (LDA) inhibitory activity on a mouse pre-adipocyte cell line (MC3T3-G2/PA6), and anti-melanin deposition activity against B16-F10 melanoma cells [1, 2, 5–7].

**Fig. 1:**
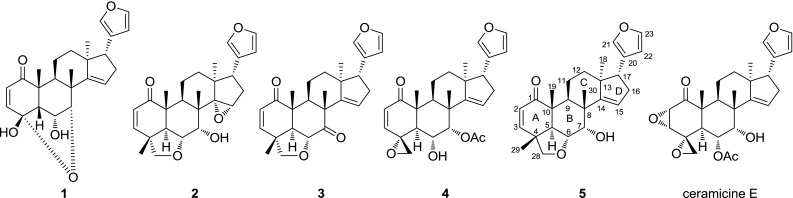
Structures of **1**–**5**

With the purpose of discovering compounds with stronger LDA inhibitory activity than ceramicine B (**5**), we further investigated the constituents of *C. ceramicus*. As a result, four new ceramicines (ceramicines M–P, **1**–**4**, Fig. 1) were isolated and their structures were determined on the basis of NMR and mass spectroscopic analyses in combination with NMR chemical shift calculations. In addition, on the basis of the LDA inhibitory activity of the isolated compounds, we further investigated the effect of the substituent at C-7 of **5**.

## Results and discussions

### Structure elucidation of ceramicines M–P (**1**–**4**)

Ceramicine M (**1**) was obtained as an optically active, [α]_D_^30^ −47 (*c* 1.0, CHCl_3_), white amorphous solid and was revealed to have the molecular formula C_24_H_28_O_5_ by HRESITOFMS [*m*/*z* 419.1823 (M + Na)^+^, Δ −1.1 mmu]. IR absorptions implied the presence of α,β-unsaturated ketone (1690 cm^−1^) and hydroxy (3350 cm^−1^) groups. ^1^H and ^13^C NMR data (Table 1) revealed 24 carbon resonances due to one carbonyl, two* sp* ^2^ quaternary carbons, four* sp* ^3^ quaternary carbons, six* sp* ^2^ methines, five* sp* ^3^ methines, three* sp* ^3^ methylenes, and three methyls. Among them, three* sp* ^3^ carbons (*δ*_C_ 73.5, 92.4, and 101.7) and two* sp* ^2^ methines (*δ*_C_ 141.1 and 143.8) were ascribed to those bearing an oxygen atom.

**Table 1:** ^1^H and ^13^C NMR data of **1**–**4**

Nos.	**1** ^a^		**2** ^b^		**3** ^b^		**4** ^b^	
	*δ*_H_ (*J*, Hz)	*δ*_C_	*δ*_H_ (*J*, Hz)	*δ*_C_	*δ*_H_ (*J*, Hz)	*δ*_C_	*δ*_H_ (*J*, Hz)	*δ*_C_
1		203.5		202.8		201.6		202.1
2	5.89 (1H, d, 10.1)	127.0	5.84 (1H, d, 9.6)	130.2	5.88 (1H, d, 9.9)	130.2	6.01 (1H, d, 10.1)	130.9
3	6.56 (1H, d, 10.1)	143.3	6.96 (1H, d, 9.6)	151.3	6.97 (1H, d, 9.9)	150.6	6.24 (1H, d, 10.1)	146.8
4		101.7		42.0		43.0		60.3
5	2.46 (1H, s)	60.0	2.94 (1H, d, 12.6)	47.4	2.26 (1H, d, 14.1)	58.7	2.83 (1H, d, 11.1)	44.9
6	4.57 (1H, s)	73.5	4.09 (1H, dd, 12.6, 2.5)	73.0	4.95 (1H, d, 14.1)	76.1	4.18 (1H, dd, 11.0, 2.5)	65.6
7	4.66 (1H, s)	92.4	3.75 (1H, br s)	71.7		205.6	5.38 (1H, d, 2.5)	75.0
8		46.5		44.2		55.3		42.6
9	2.68 (1H, dd, 13.3, 6.1)	35.5	2.63 (1H, dd, 10.7, 8.8)	35.5	2.41 (1H, m)	47.6	2.65 (1H, dd, 11.7, 5.2)	33.8
10		49.8		47.1		46.6		50.2
11a	1.21 (1H, m)	17.0	1.86 (1H, m)	18.0	1.89 (1H, m)	19.1	1.57 (1H, m)	17.9
11b	1.52 (1H, m)		2.47 (1H, m)		2.55 (1H, m)		2.46 (1H, m)	
12a	1.62 (1H, m)	33.0	1.72 (1H, m)	33.37	1.71 (1H, m)	34.7	1.60 (1H, m)	33.2
12b	1.76 (1H, br t, 11.9)		1.82 (1H, m)		1.83 (1H, m)		1.93 (1H, m)	
13		48.2		44.4		47.6		46.9
14		160.2		80.4		151.2		157.7
15	5.54 (1H, br s)	119.1	3.70 (1H, d, 2.8)	63.9	5.77 (1H, br s)	127.1	5.49 (1H, br s)	120.9
16a	2.30 (1H, ddd, 14.5, 6.7, 3.4)	35.2	1.91 (1H, dd, 14.7, 8.3)	33.43	2.40 (1H, m)	34.9	2.31 (1H, ddd, 15.4, 7.3, 3.3)	34.2
16b	2.52 (1H, dd, 14.5, 11.6)		2.27 (1H, ddd, 14.7, 9.8, 2.8)		2.58 (1H, m)		2.39 (1H, dd, 15.4, 10.6)	
17	2.79 (1H, dd, 11.6, 6.8)	54.1	3.06 (1H, dd, 9.8, 8.3)	51.0	2.84 (1H, dd, 11.1, 7.3)	51.9	2.80 (1H, dd, 10.6, 7.3)	52.0
18	0.71 (3H, s)	21.6	0.80 (3H, s)	19.3	0.78 (3H, s)	23.2	0.85 (3H, s)	21.9
19	1.18 (3H, s)	15.1	1.21 (3H, s)	14.5	1.33 (3H, s)	14.2	1.33 (3H, s)	14.8
20		125.8		125.6		124.6		124.6
21	7.29 (1H, s)	141.1	7.15 (1H, s)	139.6	7.26 (1H, s)	140.1	7.22 (1H, s)	139.6
22	6.28 (1H, s)	112.0	6.25 (1H, s)	111.3	6.28 (1H, s)	111.1	6.27 (1H, s)	111.0
23	7.39 (1H, s)	143.8	7.32 (1H, s)	142.8	7.37 (1H, s)	142.5	7.36 (1H, s)	142.5
28			3.65 (1H, d, 7.1)	80.1	3.67 (1H, d, 7.3)	79.5		
28			3.77 (1H, d, 7.1)		3.84 (1H, d, 7.3)			
29			1.33 (3H, s)	20.1	1.40 (3H, s)	19.8	3.09 (1H, d, 3.3)	50.5
29							3.64 (1H, d, 3.3)	
30	1.15 (3H, s)	24.7	1.13 (3H, s)	22.3	1.44 (3H, s)	26.6	1.23 (3H, s)	26.3
COMe								170.8
COMe							1.99 (3H, s)	21.1

^a^In CD_3_OD

^b^CDCl_3_

**Fig. 2:**
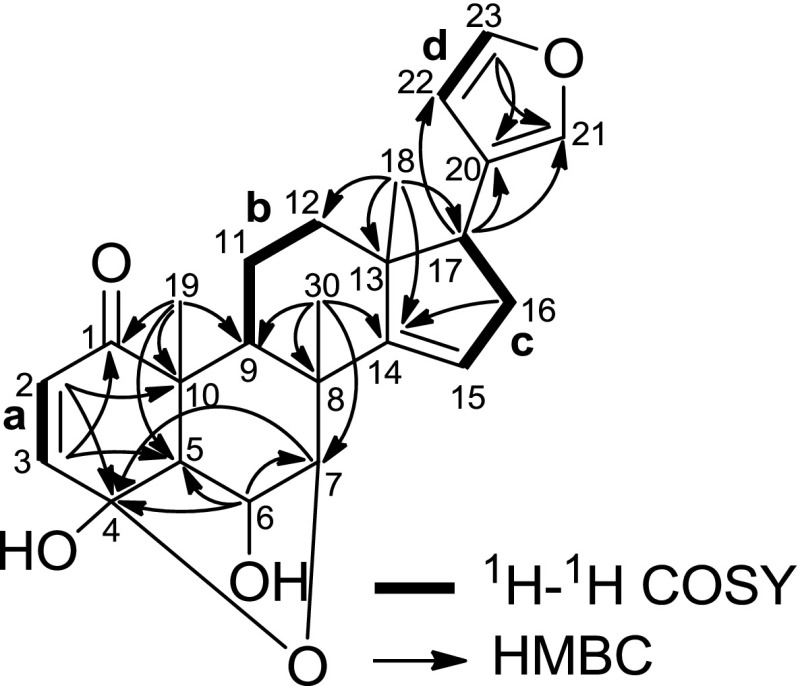
Selected 2D NMR correlations of **1**

Analyses of the HSQC and ^1^H–^1^H COSY spectra (Fig. 2) revealed the presence of four partial structures: **a** (C-2 and C-3), **b** (C-9, C-11, and C-12), **c** (C-15 to C-17), and **d** (C-22 and C-23). HMBC correlations of H_3_-18 to C-12, C-13, C-14, and C-17 suggested the connectivity of **b**, **c**, C-14, and C-18 through C-13. HMBC correlations of H-17 to C-20, C-21, and C-22, and H-23 to C-20 and C-21 suggested the presence of β-furyl at C-17, and the correlation of H_2_-16 to C-14 completed the structure of ring D. The presence of ring C was deduced from the HMBC cross-peaks of H_3_-30 to C-7, C-8, C-9, and C-14, and the connectivity of **b**, C-1, C-5, and C-19 through C-10 was suggested by the HMBC correlations of H_3_-19 to C-1, C-5, C-9, and C-10. HMBC correlations of H-2 to C-10 and C-4, and H-3 to C-1 and C-5 suggested the presence of ring A. Finally, HMBC correlations of H-6 to C-4, C-5, and C-7, and H-7 to C-4, and the chemical shift of C-4 (*δ*_C_ 101.7) suggested the planar structure of **1** to be as shown in Fig. 2.

**Fig. 3:**
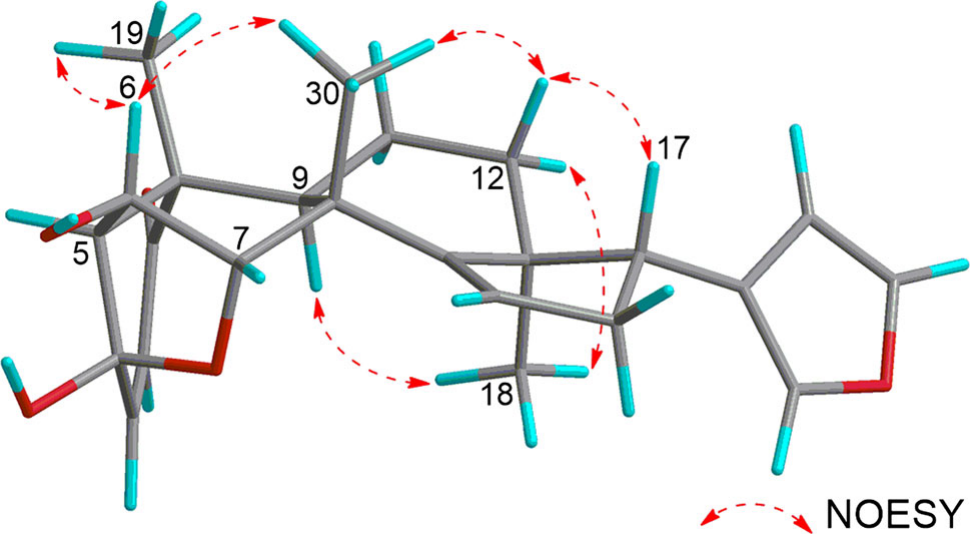
Selected NOESY correlations of **1**

The relative configuration of **1** was assigned by analyses of the ^1^H–^1^H coupling constant data and the NOESY correlations (Fig. 3). First, H-6, H-17, CH_3_-19, and CH_3_-30 were assigned to be β-axially oriented from the NOESY correlations of H-6/H_3_-19 and H_3_-30, and H-12a/H-17 and H_3_-30, while H-9 and CH_3_-18 were deduced to possess α-orientation from the NOESY correlations of H_3_-18/H-9 and H-12b. Both H-5 and H-7 should possess β-orientation since C-4 and C-7 can only be connected through an ether linkage, and the multiplicity pattern of H-6 (br s) further supports this assumption.

Ceramicine N (**2**) was obtained as an optically active, [α]_D_^31^ +50 (*c* 1.0, CHCl_3_), white amorphous solid and was revealed to have the molecular formula C_26_H_32_O_5_ by HRESITOFMS [*m*/*z* 447.2157 (M + Na)^+^, Δ +1.0 mmu]. IR absorptions implied the presence of α,β-unsaturated ketone (1680 cm^−1^) and hydroxy (3480 cm^−1^) groups. ^1^H and ^13^C NMR data (Table 1) of **2** were highly similar to those of **5** [2]. In comparison to **5**, the ^1^H and ^13^C NMR data of **2** showed an oxymethine signal (*δ*_H_ 3.70, *δ*_C_ 63.9) and* sp* ^3^ quaternary carbon (*δ*_C_ 80.4) in place of a double bond signals (*δ*_H_ 5.59, *δ*_C_ 120.4, and *δ*_C_ 159.8), and shifts of ^1^H and ^13^C signals of ring D. These data suggested **2** to be the 14,15-epoxy derivative of **5**. The presence of the 14,15-epoxy moiety in **2** was also confirmed by HMBC correlations of H_2_-16 and H_3_-30 to *δ*_C_ 80.4 (C-14) and ^1^H–^1^H COSY correlation of H-16b with *δ*_H_ 3.70 (H-15). The orientation of the epoxy group was assumed to be α on the basis of the NOESY correlation of H-15/H_3_-30 and the multiplicity of H-15 (d, 2.8 Hz). In the case of a β-oriented epoxy group, the multiplicity of H-15 would be a triplet. This assumption was further supported by DFT NMR chemical shift calculations of the two possible isomers, **2a** with α-oriented epoxy group and **2b** with β-oriented epoxy group. As can be seen in Table 2, among the two possible isomers **2a** gave the smallest mean average difference (MAD) and root-mean-square difference (RMSD) between the calculated and experimental chemical shifts, indicating the **2a** as the more likely structure.

**Table 2:** DFT calculated ^13^C NMR data of **2**

Nos.	Expt.	Calc.		Nos.	Expt.	Calc.	
		**2a**	**2b**			**2a**	**2b**
8	44.2	49.9	50.5	16	33.43	31.5	30.9
12	33.37	31.0	28.9	17	51.0	49.4	41.0
13	44.4	46.3	43.3	18	19.3	20.5	24.0
14	80.4	78.4	74.9	30	22.6	16.5	15.3
15	63.9	61.7	56.4				
MAD^a^						2.37	5.27
RMSD^a^						2.71	5.88

*MAD* mean average difference,* RMSD *root-mean-square difference

^a^Smaller is better

Ceramicine O (**3**) was obtained as an optically active, [α]_D_^30^ +10 (*c* 1.0, CHCl_3_), white amorphous solid and was revealed to have the molecular formula C_26_H_30_O_4_ by HRESITOFMS [*m*/*z* 429.2045 (M + Na)^+^, Δ +0.3 mmu]. IR absorptions implied the presence of ketone (1730 and 1680 cm^−1^) and hydroxy (3480 cm^−1^) groups. ^1^H and ^13^C NMR data (Table 1) of **3** were highly similar to those of **5** [2]. In comparison to **5**, the ^1^H and ^13^C NMR data of **3** showed a carbonyl signal (*δ*_C_ 205.6) in place of an oxymethine (*δ*_H_ 4.23, *δ*_C_ 72.5), and downfield shifts of CH-6 and C-8 signals. These data suggested **3** to be the 7-oxo derivative of **5**. The presence of a carbonyl at C-7 in **3** was also confirmed by HMBC correlations of H-5 and H_3_-30 to *δ*_C_ 205.6 (C-7). The relative configurations of **3** were deduced to be similar to those of **5** on the basis of the ^1^H–^1^H coupling constant data and NOESY correlations.

Ceramicine P (**4**) was obtained as an optically active, [α]_D_^31^ +34 (*c* 1.0, CHCl_3_), white amorphous solid and was revealed to have the molecular formula C_27_H_32_O_6_ by HRESITOFMS [*m*/*z* 475.2111 (M + Na)^+^, Δ +1.4 mmu]. IR absorptions implied the presence of ketones (1740 and 1690 cm^−1^) and hydroxy (3480 cm^−1^) groups. ^1^H and ^13^C NMR data (Table 1) of **4** were highly similar to those of ceramicine E [3]. In contrast to ceramicine E, the ^1^H and ^13^C NMR data of **4** showed two olefinic methine signals (*δ*_H_ 6.01, *δ*_C_ 130.9 and *δ*_H_ 6.24, *δ*_C_ 146.8) instead of two oxymethine signals (*δ*_H_ 3.01, *δ*_C_ 57.5 and *δ*_H_ 3.35, *δ*_C_ 52.9), indicating the presence of an α,β-unsaturated ketone in **4** in place of an α,β-epoxy ketone in ceramicine E. In addition, the chemical shifts of H-6 (*δ*_H_ 4.18) and H-7 (*δ*_H_ 5.38) of **4** indicate the presence of an acetyl at C-7. The structure of **4** was further confirmed by analyses of its 2D NMR data. In particular, HMBC correlations of H_2_-29 to *δ*_C_ 146.8 (C-3), H_3_-30 with *δ*_C_ 75.0 (C-7), and H-7 to *δ*_C_ 170.8 (COMe) confirmed the presence of an α,β-unsaturated ketone and the acetyl position. The relative configurations of **4** were deduced to be similar to those of ceramicine E on the basis of the ^1^H–^1^H coupling constant data and NOESY correlations.

The absolute configurations of the isolated compounds were assumed to be similar to those of the previously reported ceramicines. The similarities of the CD spectra of **2** and **3** with those of ceramicine B, and **4** with ceramicine E further support this assumption.

The isolated compounds were tested for LDA inhibitory activity on MC3T3-G2/PA6 cells. As can be seen in Table 3, **1**, **2**, and **4** are less potent than **5**. However, **3** showed better selectivity than **5** while showing activity at the same order of magnitude as **5**.

**Table 3:** LDA inhibitory activity and cytotoxicity

Nos.	LDA inhibitory IC_50_ (μM)	Cytotoxicity CC_50_ (μM)	Selectivity index (CC_50_/IC_50_)
**1**	11.6	29.4	2.5
**2**	7.1	>50	>7.0
**3**	3.3	>50	>15.2
**4**	>50	>50	ND
**5** ^a^	1.8	15.9	8.8
**6**	4.7	53.2	11.3
**7**	12.2	>50	>4.1
**8**	>50	>50	ND
**9**	11.6	18.3	1.6
**10**	12.0	18.9	1.6
**11**	14.8	>50	>3.4
**12** ^a,b^	8.8	26.2	3.0
**13** ^a,c^	7.7	20.6	2.7
**14** ^a,d^	>50	>50	ND

*ND *not determined

^a^Reported in Wong et al. [5]

^b^7-*epi*-**9**

^c^7-*epi*-**10**

^d^7-*epi*-**11**

### Syntheses of ceramicine B derivatives

We have previously reported that the etherification or esterification of the 7α-OH group resulted in the increase of the cytotoxicity or decrease of LDA inhibitory activity [5]. In this work, we found that **3** with a carbonyl moiety at C-7 showed better selectivity than **5** while showing activity at the same order of magnitude as **5**. Thus, we decided to further investigate the effect of the substituent at C-7 of **5**.

**Scheme 1:**
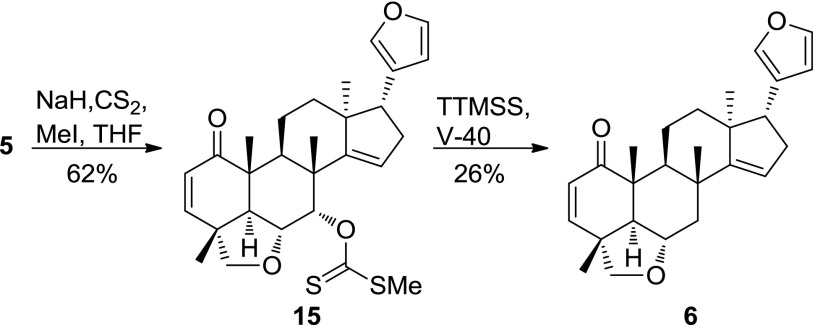
Synthesis of **6**

**Scheme 2:**
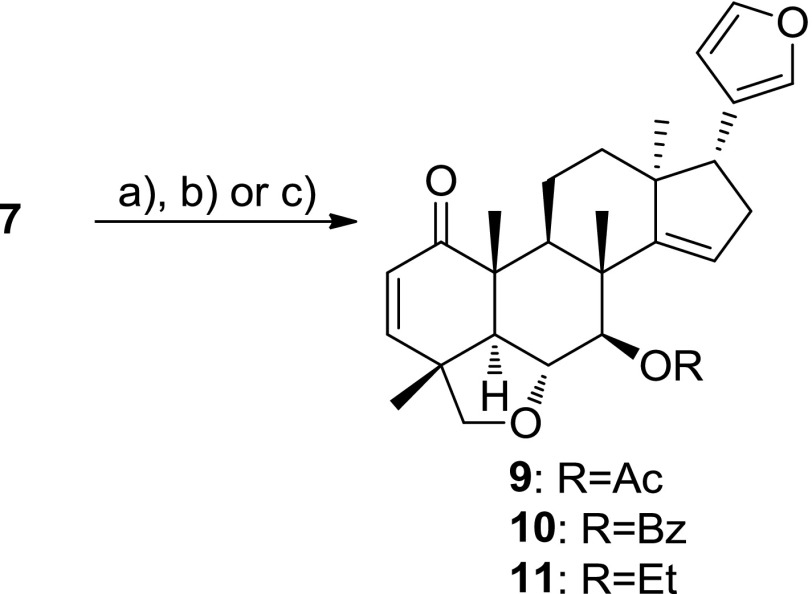
Synthesis of **9**–**11**. Reagents and conditions: *a* Ac_2_O, pyridine, DMAP, rt, 15 min, 64%; *b* BzCl, pyridine, DMAP, rt, 2 h, 32%; ***c*** EtI, NaH, DMF, rt, 4 h, 75%

First, we synthesized 7-dehydroxyceramicine B (**6**) and 7-*epi*-ceramicine B (**7**). Compound **6** was readily obtained through Barton–McCombie deoxygenation of **5** (Scheme 1) [8]. Since inversion of the configuration at C-7 could not be achieved through Mitsunobu reaction, we examined the feasibility of obtaining **7** through reduction of compound **3**, which can be easily obtained from **5** after oxidation pyridinium chlorochromate (PCC). We used the Meerwein–Ponndorf–Verley (MPV) reduction to selectively reduce the carbonyl at C-7 [9], and as can be seen in Table 4, we obtained **7**, albeit in a low yield. Interestingly, a ring-contracted side product **8** can also obtained using the MPV reduction (Table 4 entry 2). After obtaining **7**, we synthesized its derivatives **9**–**11** (Scheme 2) to study the effects of etherification and esterification of the 7β-OH on the LDA inhibitory activity.

**Table 4:** Reagents and conditions for the MPV reduction of **3**

Nos.	Reagent	Temp	Time (h)	Yield (%)		
				**5**	**7**	**8**
1	^*i*^Bu_2_AlO^*i*^Pr	70 °C	5	33	3	0
2	^*i*^Bu_2_AlO^*i*^Pr	Reflux	0.5	36	12	8
3	^*i*^Bu_2_AlOH	rt	20	36	15	0

The LDA inhibitory activities of the synthesized compounds are shown in Table 3. Compounds **6** and **7** have lower LDA inhibitory and cytotoxic activities than **5**. Thus, the absence of an α-oriented hydroxy group at C-7 led to a decrease of both the LDA inhibitory and cytotoxic activities.

The effects of esterification and etherification of 7-OH group on the LDA inhibitory activity are as follow. On the basis of the IC_50_ values of **7** and **9**–**11**, for the 7β-OH derivatives, esterification and etherification did not significantly change the LDA inhibitory activities. In contrast, on the basis of the IC_50_ values of **5** and **12**–**14**, for the 7α-OH derivatives, esterification and etherification led to lower LDA inhibitory activities.

In addition, compound **8**, with a contracted B-ring, showed no LDA inhibitory activity at 50 µM.

## Experimental section

### General experimental procedures

Optical rotations were measured on a JASCO DIP-1000 polarimeter. UV spectra were recorded on a Shimadzu UVmini-1240 spectrophotometer and IR spectra on a JASCO FT/IR-4100 spectrophotometer. High-resolution ESI MS were obtained on a LTQ Orbitrap XL (Thermo Scientific). ^1^H and 2D NMR spectra were measured on a 400-MHz or 600-MHz spectrometer at 300 K, while ^13^C NMR spectra were measured on a 100-MHz or 150-MHz spectrometer. The residual solvent peaks were used as internal standards (*δ*_H_ 7.26 and *δ*_C_ 77.0 for CDCl_3_, *δ*_H_ 3.31 and *δ*_C_ 49.0 for CD_3_OD). Standard pulse sequences were used for the 2D NMR experiments. Merck silica gel 60 (40–63 µm) was used for the column chromatography, and the separations were monitored by Merck silica gel 60 F_254_, or Merck silica gel RP C-18 F_254_ TLC plates.

### Material

The bark of *C*. *ceramicus* was collected in Terengganu, Malaysia in July 2013. The botanical identification was made by Prof. A. Hamid A. Hadi, University of Malaya. Voucher specimens (No. HOSHI13CCB) are deposited in the department of pharmacognosy Hoshi University.

### Extraction and isolation

The bark of *C*. *ceramicus* (8 kg) was extracted with MeOH to obtain 1.43 kg of extract. The MeOH extract was successively partitioned with *n*-hexane, EtOAc, *n*-BuOH, and water. The *n*-hexane-soluble materials were further separated by silica gel column chromatography (*n*-hexane/EtOAc 1:0 → 1:1, CHCl_3_/MeOH 1:0 → 0:1) to obtain 10 fractions (A–J). Fraction I was further separated with an ODS silica gel column (MeOH/H_2_O 7:3 → 1:0, acetone) to obtain six fractions (I-1 to I-6). Fraction I-2 (795 mg) was then separated by HPLC (Shiseido ODS MGII 30 × 250 mm, 75% MeOH_(aq)_ at 8.0 mL/min, UV detection at 210 nm) into nine fractions (I-2-a to I-2-i). Separation of fraction I-2-d by HPLC (Shiseido ODS MGII 4.6 × 250 mm, 50% MeCN_(aq)_ at 0.5 mL/min, UV detection at 210 nm) yielded **1** (1.1 mg, 0.00008%, *t*_R_ 30.2 min). Separation of fraction I-2-e by HPLC (Nacalai tesque Cholester 10 × 250 mm, 45% MeCN_(aq)_ at 2.0 mL/min, UV detection at 210 nm) yielded **2** (5.8 mg, 0.00041%, *t*_R_ 51.0 min). Separation of fraction I-2-h by HPLC (Nacalai tesque Cholester 10 × 250 mm, 55% MeCN_(aq)_ at 2.0 mL/min, UV detection at 210 nm) yielded **3** (1.4 mg, 0.0001%, *t*_R_ 31.2 min) and **4** (6.0 mg, 0.00042%, *t*_R_ 34.1 min).

### Ceramicine M (**1**)

White amorphous solid. [α]_D_^30^ −47° (*c* 1.0, CHCl_3_). IR (film) *ν*_max_ cm^−1^: 3350 and 1690. UV *λ*_max_ (MeOH) nm (log *ε*): 204 (4.04). CD *λ*_max_ (MeOH) nm (Δ*ε*): 295 (+0.027), 225 (−1.2), 222 (+1.8), and 203 (−7.6). ^1^H and ^13^C NMR, see Table 1. ESIMS *m/z* 419 (M + Na)^+^. HRESIMS *m/z* 419.1823 [calcd. for C_24_H_28_NaO_5_ (M + Na)^+^: 419.1834].

### Ceramicine N (**2**)

White amorphous solid. [α]_D_^31^ +50° (*c* 1.0, CHCl_3_). IR (film) *ν*_max_ cm^−1^: 3480 and 1680. UV *λ*_max_ (MeOH) nm (log *ε*): 217 (3.74). CD *λ*_max_ (MeOH) nm (Δ*ε*): 336 (−0.46), 217 (+5.0), 208 (+4.5), and 205 (+4.6) nm. ^1^H and ^13^C NMR, see Table 2. ESIMS *m/z* 447 (M + Na)^+^. HRESIMS *m/z* 447.2157 [calcd. for C_26_H_32_NaO_5_ (M + Na)^+^: 447.2147].

### Ceramicine O (**3**)

White amorphous solid. [α]_D_^30^ +10° (*c* 1.0, CHCl_3_). IR (film) *ν*_max_ cm^−1^: 3480, 1730, and 1680. UV *λ*_max_ (MeOH) nm (log *ε*): 204 (4.30). CD *λ*_max_ (MeOH) nm (Δ*ε*): 300 (−1.9), 222 (+10.6), and 205 (+4.9). ^1^H and ^13^C NMR, see Table 3. ESIMS *m/z* 429 (M + Na)^+^. HRESIMS *m/z* 429.2045 [calcd. for C_26_H_30_NaO_4_ (M + Na)^+^: 429.2042].

### Ceramicine P (**4**)

White amorphous solid. [α]_D_^31^+34° (*c* 1.0, CHCl_3_). IR (film) *ν*_max_ cm^−1^: 3480, 1740, and 1690. UV *λ*_max_ (MeOH) nm (log *ε*): 204 (3.99). CD *λ*_max_ (MeOH) nm (Δ*ε*): 336 (−1.3) and 212 (+9.5). ^1^H and ^13^C NMR, see Table 4. ESIMS *m/z* 475 (M + Na)^+^. HRESIMS *m/z* 475.2111 [calcd. for C_27_H_32_NaO_6_ (M + Na)^+^: 475.2097].

### ^13^C NMR chemical shift calculations

The conformations were obtained using Monte Carlo analysis with MMFF94 force field [10–13] and charges on Macromodel 9.1 [14]. Geometries were further optimized by using RI-*J* [15–17] approximation at the DFT [18] B3LYP/TZVP [17, 19–21] level of theory. NMR shielding constant calculations were performed on the optimized ground state geometries at the DFT B3LYP/TZVP level of theory. All DFT calculations were performed using Turbomole 7.0 [22]. The ^13^C NMR chemical shifts of the isomers were obtained by Boltzmann averaging the ^13^C NMR chemical shifts of the stable conformers.

### Synthesis of **6**

Compound** 5** (40 mg, 0.1 mmol) was dissolved in 8 mL of THF, put under argon, and cooled to 0 °C. To the solution of **5**, NaH (76 mg, 3.2 mmol) was added and the solution was stirred. After 2 h, CS_2_ (800 µL, 13.2 mmol) was added, and the solution was further stirred. After 1.5 h, the solution was returned to rt before adding MeI (400 µL, 6.4 mmol) and stirred for another 4 h. Finally, cold water was added to the reaction mixture and partitioned with Et_2_O. The Et_2_O layer was dried over anhydrous Na_2_SO_4_ before being dried under reduced pressure to afford solid residues. The residues were separated by silica gel column chromatography (*n*-hexane/EtOAc, 10:1 → 3:1) to afford **15** (30 mg, 62%).

To a solution of **15** (24 mg, 0.048 mmol) in toluene (2.0 mL) under argon at rt, tris(trimethylsilyl)silane (18 µL, 0.058 mmol) and V-40 (1.2 mg, 0.0048 mmol) was added. The mixture was then stirred for 2 h at 90 °C. The reaction mixture was dried under reduced pressure to afford solid residues. The residues were separated by silica gel column chromatography (*n*-hexane/EtOAc, 10:1 → 3:1) to afford **6** (4.9 mg, 26%).

### Compound **6**

White amorphous solid. [α]_D_^19^ +115° (*c* 0.5, CHCl_3_). IR (film) *ν*_max_ cm^−1^: 1680. UV *λ*_max_ (MeOH) nm (log *ε*): 204 (4.02). CD *λ*_max_ (MeOH) nm (Δ*ε*): 334 (−0.62), 218 (+9.5), and 203 (+5.1). ^1^H NMR (400 MHz, CDCl_3_) *δ*: 0.80 (3H, s), 1.14 (3H, s), 1.18 (3H, s), 1.35 (3H, s), 1.40 (1H, t, 11.8), 1.63 (1H, m), 1.80 (1H, m), 1.88 (1H, m), 1.92 (1H, d, 11.8), 2.06 (1H, dd, 11.5, 6.3), 2.32 (1H, ddd, 15.2, 7.3, 3.5), 2.45 (1H, m), 2.49 (1H, m), 2.53 (1H, dd, 11.8, 4.5), 2.82 (1H, dd, 10.7, 7.4), 3.62 (1H, d, 7.1), 3.74 (1H, d, 7.1), 4.09 (1H, td, 11.8, 4.5), 5.49 (1H, br s), 5.82 (1H, d, 9.7), 6.29 (1H, s), 6.95 (1H, d, 9.7), 7.24 (1H, s), 7.37 (1H, s). ^13^C NMR (100 MHz, CDCl_3_) *δ*: 14.5, 18.5, 20.6, 21.8, 27.5, 33, 34.2, 41.2, 42.2, 42.3, 45.9, 47.2, 47.4, 51.9, 58.5, 71.8, 79.1, 111.1, 119.2, 124.8, 129.9, 139.7, 142.5, 151.3, 162.9, 203.2. HRESIMS *m/z* 415.2241 [calcd. for C_26_H_32_NaO_3_ (M + Na)^+^: 415.2249].

### Synthesis of **3**

Compound **5** (100 mg, 0.25 mmol) was dissolved in 10 mL of CH_2_Cl_2_, put under argon, and cooled to 0 °C. To the solution of **5**, PCC (181 mg, 0.84 mmol) was added, and the solution was stirred at rt. After 2 h, more PCC (125 mg, 0.58 mmol) was added, and the solution was further stirred at rt for 2 h. To the resulting mixture, diethyl ether was added, and the solids were filtered through Celite. The filtrates were then dried under reduced pressure to afford solid residues. The residues were separated by silica gel column chromatography (*n*-hexane/EtOAc = 1:1) to afford **3** (70 mg, 70%).

### Preparation of ^*i*^Bu_2_AlO^*i*^Pr

^*i*^Bu_2_AlH (1.0 M in toluene, 5.0 mmol, 5.0 mL) was put under argon and cooled to 0 °C. Isopropanol (390 µL, 5.0 mmol) was then added, and the mixture was stirred for 1 h at rt before being used in the reactions below.

### Preparation of ^*i*^Bu_2_AlOH

^*i*^Bu_2_AlH (1.0 M in toluene, 5.0 mmol, 5.0 mL) was put under argon and cooled to 0 °C. Water (90 µL, 5.0 mmol) was then added, and the mixture was stirred for 1 h at rt before being used in the reaction below.

### Table 4: entry 1

To a solution of **3** (5 mg, 0.012 mmol) in toluene (0.5 mL) under argon, ^*i*^BuAlO^*i*^Pr (72 µL, 0.072 mmol) was added at rt. The mixture was then stirred at 70 °C for 5 h, and cooled to rt before adding 72 µL of water and stirred for another hour. To the resulting mixture, EtOAc was added, and the solids were filtered through Celite. The filtrates were then dried under reduced pressure to afford solid residues. The residues were separated by ODS HPLC (Shiseido ODS MGII 4.6 × 250 mm, MeOH/H_2_O, 75:25 at 0.5 mL/min, UV detection at 210 nm) to afford **5** (1.7 mg, 33%, *t*_R_ = 28.0 min) and **7** (0.15 mg, 3%, *t*_R_ = 26.4 min).

### Table 4: entry 2

To a solution of **3** (5 mg, 0.012 mmol) in toluene (0.5 mL) under argon, ^*i*^BuAlO^*i*^Pr (24 µL, 0.024 mmol) was added at rt. The mixture was then refluxed for 0.5 h, and cooled to rt before adding 72 µL of water and stirred for another hour. To the resulting mixture, EtOAc was added, and the solids were filtered through Celite. The filtrates were then dried under reduced pressure to afford solid residues. The residues were separated by ODS HPLC (Shiseido ODS MGII 4.6 × 250 mm, MeOH/H_2_O, 75:25 at 0.5 mL/min, UV detection at 210 nm) to afford **5** (1.8 mg, 36%, *t*_R_ = 28.0 min), **7** (0.6 mg, 12%, *t*_R_ = 26.4 min), and **8** (0.4 mg, 8%, *t*_R_ = 29.6 min).

### Table 4: entry 3

To a solution of **3** (20 mg, 0.049 mmol) in toluene (2.0 mL) under argon, ^*i*^BuAlOH (390 µL, 0.39 mmol) was added and stirred at rt. After 20 h, the mixture was cooled to 0 °C before adding 1 N HCl (5 mL). The resulting mixture was then partitioned with EtOAc, and the EtOAc layer was dried with anhydrous Na_2_SO_4_ before being dried under reduced pressure to afford solid residues. The residues were separated by preparative TLC (benzene/EtOAc, 4:1) to afford **5** (7.2 mg, 36%) and **7** (3.0 mg, 15%).

### Compound **7**

White amorphous solid. [α]_D_^20^ +43° (*c* 0.1, CHCl_3_). IR (film) *ν*_max_ cm^−1^: 3730 and 1680. UV *λ*_max_ (MeOH) nm (log *ε*): 203.5 (3.95). CD *λ*_max_ (MeOH) nm (Δ*ε*): 341 (−0.59), 219 (+6.0), and 206 (+3.7). ^1^H NMR (400 MHz, CDCl_3_) *δ*: 0.83 (3H, s), 1.18 (3H, s), 1.19 (3H, s), 1.34 (3H, s), 1.63 (1H, m), 1.76 (1H, m), 1.82 (1H, m), 2.01 (1H, d, 12.0), 2.40 (2H, m), 2.53 (1H, m), 2.88 (1H, m), 3.43 (1H, dd, 8.0, 3.2), 3.63 (1H, d, 8.0), 3.79 (1H, d, 8.0), 4.07 (1H, dt, 12.0, 8.0), 5.85 (1H, d, 11.0), 5.92 (1H, br s), 6.30 (1H, s), 6.94 (1H, d, 11.0), 7.25 (1H, s), 7.37 (1H, s). ^13^C NMR (100 MHz, CDCl_3_) *δ*: 14.8, 19.7, 19.8, 20.0, 24.7, 34.6, 37.1, 42.3, 45.9, 46.3, 47.3, 47.6, 51.7, 55.2, 77.6, 79.4, 80.9, 111.0, 124.5, 125.1, 129.8, 139.7, 142.6, 151.2, 158.1, 202.6. HRESIMS *m/z* 431.2192 [calcd. for C_26_H_32_NaO_4_ (M + Na)^+^: 431.2198].

### Compound **8**

White amorphous solid. [α]_D_^22^ +58° (*c* 0.1, CHCl_3_); IR (film) *ν*_max_ cm^−1^: 3730 and 1680. UV *λ*_max_ (MeOH) nm (log *ε*): 204.5 (3.85). CD *λ*_max_ (MeOH) nm (Δ*ε*): 336 (−0.44), 289 (−0.11), 243 (−0.49), and 202 (+5.3). ^1^H NMR (400 MHz, CDCl_3_) *δ*: 0.94 (3H, s), 1.09 (3H, s), 1.23 (3H, s), 1.27 (3H, s), 1.73 (1H, m), 1.94 (1H, m), 2.14 (1H, m), 2.33 (1H, m), 2.83 (1H, m), 2.5 (1H, m), 2.61 (1H, d, 12.0), 2.84 (1H, m), 3.42 (1H, d, 12.0), 3.69 (1H, d, 12.0), 3.75 (1H, dd, 12.0, 3.2), 4.45 (1H, dd, 12.0, 3.2), 5.8 (1H, br s), 5.89 (1H, d, 10.0), 6.28 (1H, s), 6.46 (1H, d, 10.0), 7.25 (1H, s), 7.38 (1H, s). ^13^C NMR (100 MHz, CDCl_3_) *δ*: 16.0, 16.4, 16.6, 23.4, 32.8, 33.0, 34.3, 43.2, 44.5, 44.6, 49.7, 49.9, 50.4, 52.7, 61.4, 70.3, 111.3, 120.3, 127.8, 132.5, 140.2, 142.9, 156.2, 162.5, 207.6. HRESIMS *m/z* 433.2567 [calcd. For C_26_H_32_NaO_4_ (M + Na)^+^: 433.2555].

### Synthesis of **9**

To a solution of **6** (3.0 mg, 0.007 mmol) in pyridine (0.5 mL) under argon at rt, acetic acid anhydride (1.3 µL, 0.014 mmol) and 4-dimethylaminopyridine (0.085 mg, 0.0007 mmol) were added. The mixture was then stirred for 15 min and dried under reduced pressure to afford solid residues. The residues were separated by preparative TLC (benzene/EtOAc, 4:1) to afford **9** (1.8 mg, 64%).

### Compound **9**

White amorphous solid. [α]_D_^21^ +28° (*c* 0.1, CHCl_3_). IR (film) *ν*_max_ cm^−1^: 1740 and 1680. UV *λ*_max_ (MeOH) nm (log *ε*): 203.5 (4.78). *λ*_max_ (MeOH) nm (Δ*ε*): 334 (−4.3) and 223 (+43). ^1^H NMR (400 MHz, CDCl_3_) *δ*: 0.86 (3H, s), 1.19 (3H, s), 1.25 (3H, s), 1.33 (3H, s), 1.62 (1H, m), 1.76 (1H, m), 1.84 (1H, m), 2.11 (1H, d, 12.0), 2.15 (3H, s), 2.32 (2H, m), 2.54 (1H, m), 2.84 (1H, m), 3.67 (1H, d, 7.2), 3.75 (1H, d, 7.2), 4.20 (1H, dd, 12.0, 8.8), 4.92 (1H, d, 8.8), 5.55 (1H, br s), 5.85 (1H, d, 9.6), 6.29 (1H, s), 6.93 (1H, d, 9.6), 7.22 (1H, s), 7.36 (1H, s). ^13^C NMR (100 MHz, CDCl_3_) *δ*: 15.1, 19.9, 20.0, 20.4, 21.5, 24.9, 34.8, 37.9, 42.2, 45.9, 46.7, 47.6, 51.8, 55.5, 75.3, 79.6, 80.7, 111.2, 124.4, 125.5, 129.9, 139.8, 142.7, 151.3, 156.0, 170.3, 202.3. HRESIMS *m/z* 473.2313 [calcd. for C_28_H_34_NaO_5_ (M + Na)^+^: 473.2304].

### Synthesis of **10**

To a solution of **6** (3.0 mg, 0.007 mmol) in pyridine (0.5 mL) under argon at rt, benzoyl chloride (1.7 µL, 0.015 mmol) and 4-dimethylaminopyridine (0.085 mg, 0.0007 mmol) were added. The mixture was then stirred for 2 h and dried under reduced pressure to afford solid residues. The residues were separated by preparative TLC (benzene/EtOAc, 4:1) to afford **10** (1.1 mg, 32%).

### Compound **10**

White amorphous solid. [α]_D_^19^ +81° (*c* 0.1, CHCl_3_). IR (Film) *ν*_max_ cm^−1^: 1720 and 1680. UV *λ*_max_ (MeOH) nm (log *ε*): 225 (4.19) and 201.5 (4.20). CD *λ*_max_ (MeOH) nm (Δ*ε*): 329 (−0.81), 227 (+11), and 211 (+6.2). ^1^H NMR (400 MHz, CDCl_3_) *δ*: 0.91 (3H, s), 1.24 (3H, s), 1.35 (3H, s), 1.40 (3H, s), 1.65 (1H, m), 1.75 (1H, m), 1.84 (1H, m), 2.20 (1H, d, 12.0), 2.22 (2H, m), 2.58 (1H, m), 2.82 (1H, m), 3.71 (1H, d, 7.6), 3.76 (1H, d, 7.6), 4.34 (1H, dd, 12.0, 9.6), 5.21 (1H, d, 9.6), 5.49 (1H, br s), 5.87 (1H, d, 10.0), 6.28 (1H, s), 6.96 (1H, d, 10.0), 7.20 (1H, s), 7.35 (1H, s), 7.47 (2H, t, 7.6), 7.58 (1H, t, 7.6), 8.14 (2H, d, 7.6); ^13^C NMR (100 MHz, CDCl_3_) *δ*: 14.9, 20.0, 20.9, 24.7, 37.4, 42.3, 46.0, 47.6, 51.6, 55.6, 75.3, 79.6, 81.2, 111.0, 124.5, 125.7, 128.6, 130.0, 133.2, 139.8, 142.7, 151.2, 156.1, 165.7, 185.2, 202.4. HRESIMS *m/z* 535.2452 [calcd. for C_33_H_36_NaO_5_ (M + Na)^+^: 535.2460].

### Synthesis of **11**

To a solution of **6** (1.5 mg, 0.004 mmol) in DMF (0.5 mL) under argon at rt, EtI (5.7 µL, 0.018 mmol) and NaH (0.4 mg, 0.018 mmol) were added, and the mixture was then stirred. After 4 h, saturated NH_4_Cl was added, and the resulting mixture was then partitioned with EtOAc. The EtOAc layer was dried with anhydrous Na_2_SO_4_ before being dried under reduced pressure to afford solid residues. The residues were separated by preparative TLC (benzene/EtOAc, 4:1) to afford **11** (1.3 mg, 75%).

### Compound **11**

White amorphous solid. [α]_D_^22^+31° (*c* 0.1, CHCl_3_). IR (Film) *ν*_max_ cm^−1^: 1680. UV *λ*_max_ (MeOH) nm (log *ε*): 203.5 (3.70). CD *λ*_max_ (MeOH) nm (Δ*ε*): 332 (−0.25), 224 (+2.4), and 202 (+1.2). ^1^H NMR (400 MHz, CDCl_3_) *δ*: 0.84 (3H, s), 1.16 (3H, s), 1.19 (3H, s), 1.24 (3H, t, 6.8), 1.32 (3H, s), 1.66 (1H, m), 1.78 (1H, m), 2.38 (2H, m), 2.46 (1H, m), 2.76 (1H, d, 12.0), 2.84 (1H, m), 3.54 (2H, q, 6.8), 3.60 (1H, d, 7.2), 3.76 (1H, d, 7.2), 4.10 (1H, dd, 12.0, 2.8), 5.83 (1H, d, 10.0), 5.98 (1H, br s), 6.31 (1H, s), 6.94 (1H, d, 10.0), 7.25 (1H, s), 7.37 (1H, s). ^13^C NMR (100 MHz, CDCl_3_) *δ*: 14.7, 15.6, 19.6, 20.2, 20.8, 24.3, 34.7, 36.4, 41.9, 45.5, 46.2, 46.5, 47.5, 51.7, 55.3, 67.6, 78.0, 79.1, 88.7, 111.1, 124.9, 129.8, 139.6, 142.5, 151.3, 157.4, 202.8. HRESIMS *m/z* 459.2514 [calcd. for C_28_H_36_NaO_4_ (M + Na)^+^: 459.2511].

### LDA inhibitory activity and cytotoxicity

MC3T3-G2/PA6 murine pre-adipocytes (Riken Cell Bank, Ibaraki, Japan) were maintained in basal medium [alpha minimum essential medium (α-MEM) (Wako, Osaka, Japan) supplemented with 10% FBS (Cell Culture Bioscience, Tokyo, Japan)]. LDA inhibitory activity and cytotoxicity on MC3T3-G2/PA6 were measured using the same methods as in our previous report [5, 6]. Briefly, the LDA inhibitory activity of the samples was measured on the basis of the amount of LDA after 6 days of incubation with a mixture of 3-isobutyl-1-methylxanthine (IBMX), dexamethasone (DEX), and insulin (MDI inducer), and was expressed as IC_50_ value (the concentration of the sample causing 50% inhibition of LDA relative to an untreated control). The cytotoxicity was evaluated indirectly via MTT assay which is based on mitochondrial succinate dehydrogenase activity and confirmed via microscopic observation. The cytotoxicity was expressed as CC_50_ value which was defined as the concentration of the sample causing 50% cell viabilities relative to an untreated control.
